# A comparative study of genome organization and inferences for the systematics of two large bushcricket genera of the tribe Barbitistini (Orthoptera: Tettigoniidae: Phaneropterinae)

**DOI:** 10.1186/1471-2148-14-48

**Published:** 2014-03-13

**Authors:** Beata Grzywacz, Dragan P Chobanov, Anna Maryańska-Nadachowska, Tatyana V Karamysheva, Klaus-Gerhard Heller, Elżbieta Warchałowska-Śliwa

**Affiliations:** 1 Institute of Systematics and Evolution of Animals Polish Academy of Sciences, Sławkowska 17, Krakow 31-016, Poland; 2 Institute of Biodiversity and Ecosystem Research, Bulgarian Academy of Sciences, Sofia, Bulgaria; 3 Institute of Cytology and Genetics of the Siberian Branch of the Russian Academy of Sciences, Novosibirsk, Russia; 4 Grillenstieg 18, Magdeburg 39120, Germany

**Keywords:** FISH, rDNA, Heterochromatin, Evolution, Orthoptera, *Poecilimon*, *Isophya*

## Abstract

**Background:**

*Poecilimon* and *Isophya* are the largest genera of the tribe Barbitistini and among the most systematically complicated and evolutionarily intriguing groups of Palearctic tettigoniids. We examined the genomic organization of 79 taxa with a stable chromosome number using classical (C–banding, silver and fluorochrome staining) and molecular (fluorescence *in situ* hybridization with 18S rDNA and (TTAGG)_*n*_ telomeric probes) cytogenetic techniques. These tools were employed to establish genetic organization and differences or similarities between genera or species within the same genus and determine if cytogenetic markers can be used for identifying some taxonomic groups of species.

**Results:**

Differences between the karyotypes of the studied genera include some general changes in the morphology of the X chromosome in *Isophya* (in contrast to *Poecilimon*). The number of major rDNA clusters per haploid genome divided *Poecilimon* into two main almost equal groups (with either one or two clusters), while two rDNA clusters predominated in *Isophya*. In both genera, rDNA loci were preferentially located in the paracentromeric region of the autosomes and rarely in the sex chromosomes. Our results demonstrate a coincidence between the location of rDNA loci and active NORs and GC-rich heterochromatin regions. The C/DAPI/CMA_3_ bands observed in most *Poecilimon* chromosomes suggest the presence of more families of repetitive DNA sequences as compared to the heterochromatin patterns in *Isophya*.

**Conclusions:**

The results show both differences and similarities in genome organization among species of the same genus and between genera. Previous views on the systematics and phylogenetic grouping of certain lineages are discussed in light of the present cytogenetic results. In some cases, variation of chromosome markers was observed to correspond with variation in other evolutionary traits, which is related to the processes of ongoing speciation and hybridization in zones of secondary contact. It was concluded that the physical mapping of rDNA sequences and heterochromatin may be used as an additional marker for understanding interspecific relationships in these groups and their routes of speciation.

## Background

*Isophya* and *Poecilimon* represent the most evolutionarily successful genera of the tribe Barbitistini (sometimes regarded as a subfamily), accounting for about 82% species of this taxon. Both genera represent herbivorous short-winged bushcrickets with complex acoustic behavior [1]. Their center of diversification and, possibly, origin, is the Pontic region, and especially Anatolia, which is home to a vast number of taxa (a total of 136 *Poecilimon* species and 86 *Isophya* species; [2,3]) with restricted ranges [4,5]. A great number of taxa are local endemics; nevertheless, they often occur in huge numbers and thus may damage crops [6,7].

Several attempts to classify these two groups have been made, mainly on a smaller scale (within genera) and using various approaches, such as morphology [6,8,9], bioacoustic and morphological traits [5,10-17], cytogenetic data [18-20], and molecular phylogenies [21,22]. Yet, many questions and doubts remain unanswered due to the large number of taxa, the vast recent radiation in some lineages and, possibly, the considerable contribution of present and past hybridization events [5,20].

Ribosomal DNA genes (rDNA) and active nucleolus organizer regions (NORs) are very useful chromosome markers for interspecific comparisons. Over the last few years, fluorescence *in situ* hybridization (FISH) techniques have been extensively used for understanding the karyotype structure and evolution of various insects, especially coleopterans (e.g. Scarabaeinae [23]), lepidopterans [24], and orthopterans (e.g. Acrididae grasshoppers) [25-30]. In other Orthoptera, e.g. tettigoniids, repeated DNA has been analyzed by silver impregnation (Ag-NORs) and chromosomal mapping with a view to location of the major ribosomal DNA cluster (18S rDNA probe) and telomere repeats (TTAGG)_*n*_ in the subfamilies Saginae [31,32], Bradyporinae [33], and Phaneropterinae [20,34-36].

To date, the chromosomal organization (i.e. the distribution of heterochromatin and the location of 18S loci and active NORs) of Barbitistini was analyzed in species belonging to eight genera of this tribe. In these lineages the latter markers proved to be good for understanding genomic differentiation and distinguishing between species and evolutionary lines [37]. In this work, we present a detailed cytogenetic analysis of 79 taxa belonging to the genera *Poecilimon* and *Isophya*. We examined their chromosomal characteristics, the number and distribution of major rDNA clusters using FISH with an 18S rDNA probe, and active NOR locations by silver staining. In FISH experiments, we also used the telomeric (TTAGG)_*n*_ probe for better identification of chromosome ends. In addition, some *Isophya* species/subspecies/populations for which rDNA cluster data were previously available [20] were included in our analysis. Furthermore, conventional methods, such as C-banding and fluorochrome CMA_3_ and DAPI staining, were used to analyze heterochromatin composition in representatives of both genera. The main objectives of the present work were to test (1) how the structure and distribution of rDNA clusters and heterochromatin affect the genomic organization of *Poecilimon* and *Isophya*, (2) whether the general tendencies in chromosomal organization correspond to morphoacoustic specializations, and (3) whether these tendencies reflect recent concepts concerning the systematics and phylogeny of both genera.

## Results

All analyzed *Poecilimon* and *Isophya* species showed a 2n = 31 karyotype in the males and a 2n = 32 karyotype in the females with an X0/XX sex determination system. Acrocentric autosomes were divided into two groups: four long (1–4) and eleven medium or short ones (5–15), both of which gradually decreased in size and sometimes minor length differences in chromosome pairs might cause problems with their precise identification. Combining the results of all cytogenetic markers allowed for the identification of homologous chromosome targets, yet sometimes the ordering of particular pairs could be imprecise. In most *Poecilimon* species, the X chromosome was acrocentric, except for subacrocentric chromosomes in *P. jonicus tessellatus*, *P. martinae*, and *P. macedonicus* (Table 1). Similarly to the results published previously [19], most *Isophya* species analyzed in this study exhibited a subacrocentric X (Table 2).

**Table 1:** ***Poecilimon *** **species: collection localities, sex chromosome types (X), and chromosomal location of rDNA clusters**

	**Species**	**Collection sites**	**X**	**rDNA-FISH signal**	
				**Localization**	**Total**
**1**	*P. ledereri* Ramme, 1933	TR, Izmir prov., Izmir	a	11/13p	1
	*P.* aff. *ledereri* Ramme, 1933	TR, Karabük prov., Agaçkesen Köyü	a	11/12p	1
	*P. orbelicus* Pancic, 1883	BG, Blagoevgrad distr., Rila Mts, Eleshnitsa	a	11/12p	1
	*P. armeniacus* (Uvarov, 1921)	TR, (1) Tokat/Sivas prov. border, Çamlibel pass;	a	(1) 9/10p;	1
		(2) Agri prov., Balik Gölü		(2) 11/13p	1
	*P. ampliatus* Brunner von Wattenwyl, 1878	SL, Slavnik Mt.	a	2/3, 9/10p	2
	*P. pechevi* Andreeva, 1978	BG, Blagoevgrad distr., Vlahina Mt., Kadiytsa Peak	a	3/4p, 12/13p	2
	*P. ebneri* Ramme, 1933	MK, Mariovo Region, W of Skochivir vill.	a	3/4*p, 12/13p	2
	*P. klisuriensis* Willemse, 1982	MK, Baba (Pelister) Mt., Gjavato pass	a	5p, 11p	2
	*P. marmaraensis* Naskrecki, 1991	BG, Sliven distr., E Stara Planina Mt.	a	4/5p, Xp	2
**2**	*P.* cf. *karakushi* Ünal, 2003	TR, Isparta prov., S Taurus Mts, Davras Resort	a	11/13p	1
	*P. ersisi* Salman, 1978	TR, Tokat/Sivas prov. border, Çamlibel pass	a	11/13p	1
	*P. serratus* Karabag, 1962	TR, Bursa prov., Keles	a	11/12p	1
	*P. toros* Ünal, 2003	TR, Antalya	a	12/13p	1
**3**	*P. cervus* Karabag, 1950	TR, Düzce prov., above Yıgılca	a	9p	1
	*P. pliginskii* Miram, 1929	UA, S Crimea, Chetyr Dag Mt.	a	10/11p	1
	*P.* aff. *bischoffi* Ramme, 1933	UA, S Crimea, Angarskiy Pereval Pass	a	10/11p	1
	*P. bischoffi* Ramme, 1933	TR, Rize prov., Ikizdere	a	11/13*p	1
	*P. bosphoricus* Brunner von Wattenwyl, 1878	TR, Kastamonu prov., Ilgaz Mt., Tosya pass	a	10p	1
	*P. miramae* Ramme, 1933	BG, Haskovo distr., E Rhodope Mts, Mandritsa vill.	a	9/10p	1
	*P. heinrichi* (Ramme, 1951)	BG, Bourgas distr., Strandza Mts, near Malko Tarnovo	a	12/13p	1
	*P. roseoviridis* Chobanov & Kaya, 2012	BG, (1) Bourgas distr., Kovach site; (2) Malko Tarnovo	a	3/4p, 9/11p	2
	*P. turcicus* Karabag, 1950	TR, Kırklareli prov., Mandraköy vill.	a	3/4p, 11/13p	2
	*P. anatolicus* Ramme, 1933	TR, Tekirdag prov., Elmali vill.	a	3/4*p, 11/13p	2
	*P. similis proximus* Ünal, 2010	TR, Düzce prov., near Yigilca	a	4p, 12/13p	2
**4**	*P. chopardi* Ramme, 1933	MK, Nidzhe Mt., above Skochivir vill.	a	5p, 13p	2
**5**	*P. brunneri* (Frivaldsky, 1867)	GR, Thrakien Rodopi; BG, Bourgas, Malko Tarnovo	a	11/13p	1
	*P. ukrainicus* Bey-Bienko, 1954/*fussii* Fieber,1878	BG, Rousse distr., Byala & Ivanovo vill.	a	5p, Xp	2
	*P. macedonicus* Ramme, 1926	GR, Kalambaca; MK, Nidzhe Mt., above Skochivir vill.	sa	13p, Xd	2
	*P. zwicki* Ramme, 1939	BG, Blagoevgrad distr., Maleshevska Planina Mt.	a	2d*, 5d, 7p, 9d	4
**6**	*P. jonicus tessellatus* (Fischer, 1853)	GR, Peloponnes	sa	12/13i	1
	*P. martinae* Heller, 2004	TR, Antalya	sa	11/12p	1
**7**	*P. schmidtii* (Fieber, 1853)	BG, (1) Varna distr., St.St. Konstantin and Elena Resort - Botanical Garden; (2) Vidin distr., Belogradchik	a	3/4p, 6/7p	2
	*P. zonatus* Bolívar,1899*/varicornis* (Haan,1842)	TR, Van prov., Kuskunkıran pass	a	4/5p, 10/13p	2
**8**	*P. jablanicensis* Chobanov & Heller, 2010	MK, Jablanica Mt., above Gorna Belica vill.	a	3/4*p, 7p	2
	*P. ornatus* (Schmidt, 1850)	SL, Slavnik/Garbice	a	3/4p, 12/13p	2
	*P. affinis* (Frivaldsky, 1867)	BG, (1) Kyustendil distr., Rilski Manastir; (2) Vratsa distr., Vrachanska Planina Mt.	a	2p, 5p, 7p, 13p	4
**?**	*P. ataturki* Ünal, 1999	TR, Kastamonu prov., Dеvrekani towards Yaraligöz Pass	a	12/13p	1
**?**	*P. celebi* Karabag, 1953	TR, Kastamonu prov., Dеvrekani towards Yaraligöz Pass	a	2/3p, 11/13p	2
**?**	*P.* aff *glandifer* Karabag, 1950	TR, Izmir prov., 18 km N of Ödemiş	a	3/4i-d*, 10/12p	2

BG = Bulgaria, MK = Macedonia, SL = Slovenia, GR = Greece, TR = Turkey, UA = Ukraine; X-type: a = acrocentric, sa = subacrocentric; total = number of clusters in haploid genome; a slash between two numbers indicates imprecise identification of the chromosome pair (bivalent); p = paracentromeric; d = distal; i = interstitial; i-d = interstitial near the distal end; X = sex chromosome; *high or low activity between homologous chromosomes. The first column shows groups of species/phylogenetic lineages as suggested in published sources and/or inferred from unpublished own data: 1 – node C according to Ullrich *et al.*[22] or *P. ampliatus* group s.l. see [10]; 2 – *P. syriacus* group [14]; 3 – *P. bosphoricus* group [17]; 4 – *P. propinquus* group [10]; 5 – node A according to Ullrich *et al.*[22] or *P. brunneri* group [10] s.l.; 6 – possibly belonging to a monophyletic lineage [22]; 7 – possibly belonging to a monophyletic lineage [22]; 8 – *P. ornatus* group [16]; ? – taxa with a doubtful phylogenetic position.

**Table 2:** ***Isophya *** **species: collection localities, sex chromosome types (X), and chromosomal location of rDNA clusters**

	**Species**	**Collection sites**	**X**	**rDNA-FISH signal**	
				**Localization**	**Total**
**1**	*I. hospodar* (Saussure, 1898)	BG, Sofia distr., Beledie Han	sa	3p, 10/12p	2
	*I. straubei* ssp.	TR, Isparta prov., S Taurus Mts, Davraz Resort	sa	3d*, 6p	2
**2**	*I. nervosa* Ramme, 1931	TR, Kütahya prov., E of Tavşanlı	a	3p	1
	*I. thracica* Karabag, 1962	TR, Tekirdag prov., Elmalı vill.	sa	13*p	1
	*I. stenocauda stenocauda* Ramme, 1951	TR, Karabük prov.,Agaçkesen Köyü	sa	10/12p	1
	*I. stenocauda obenbergeri* Mařan, 1958	TR, Kastamonu prov., Tosya pass	sa	3p, 11/12*p	2
	*I. rectipennis* Brunner von Wattenwyl, 1882	BG, Rousse distr., Byala;	sa	7*p, Xd;	2
		TR, Düzce prov., above Yıgılca;	a	3p, 12/13*p;	2
		►BG, Sliven distr., E Stara Planina Mts, Karandila	a	3/4p, 5d	2
	*I. pavelii* Brunner von Wattenwyl, 1882►	BG, Strandzha Mts	a	2p, 3/4*p, Xp	3
**3**	*I. bureschi* Peshev, 1981 ►	BG, (1) Blagoevgrad distr., Rila Mts, Jundola vill;	sa	(1) 3/4p, 11/12p;	(1) 2
		(2) Pazardzhik distr., Sredna Gora Mts, Oborishte hut;		(2) 11/12p;	(2) 1
		(3) Blagoevgrad distr., Pirin Mts, Gotse Delchev lodge;		(3) 3/4*p, 11/12p;	(3) 2
		(4) Sofia distr., near Plana vill.		(4) 11/12p or 4p, 11/12p	(4) 1/2
	*I. andreevae* Peshev, 1981►	BG, Blagoevgrad distr., Kresna Gorge	sa	3/4p, 10/11p	2
	*I. miksici* Peshev, 1985►	BG, (1) Vratsa distr., Vrachanska Planina Mt., Gorski Dom hotel; (2) Danubian Plane	sa	2/3p, 12/13p	2
	*I. plevnensis* Peshev, 1985►	BG, Lovech distr., Apriltsi	sa	2/3p 12/13 p	2
	*I. longicaudata adamovici* Peshev, 1985►	BG, Sliven distr., E Stara Planina Mts, Karandila	sa	3/4p, 12/13*p	2
	*I. longicaudata longicaudata* Ramme 1951►	BG, (1) Varna distr., St.St. Konstantin and Elena resort; (2) Silistra distr., Balik and Pchelnik vills	sa	3/4p, 12/13p	2
	*I. rhodopensis leonorae* Kaltenbach, 1965►	BG, Blagoevgrad distr., Alibotush Mt, Livade site	sa	2/3p, 3/4p, 6/7p, 8p, 12/13p	5
	*I. rh. petkovi* Peshev, 1959►	BG, (1) East Rhodope Mts, Perperikon site near Murgovo vill.; (2) E Rhodope Mts, Gluhite Kamani site	sa	3/4p, 5/6p, 7/8p, 12/13p	4
	*I. rh.. rhodopensis* Ramme 1951►	BG, Smolyan distr., Rodope Mts, above Smolyan	sa	3/4p, 5p, 6*p, 8/9p, 12/13p;	5
				3/4p, 5p, 8/9p,12/13	4
	*I. rh. rhodopensis / I. rh. leonorae* Kaltenbach, 1965 – intermediate/hybrid populations►	BG, (1), Smolyan distr., W Rhodope Mts, Trigrad vill.; (2) Smolyan, W Rodope Mts, near Shiroka Polana Lake; (3) Smolyan distr., W Rhodope Mts, Trigrad-Zhrebevo vills	sa	(1) 3/4p, 5/6p, 12/13*p;	(1) 3
				(2) 3/4p, 12/13p; (2) & (3)	(2) 2
				3/4*p, 5*p, 6/7*p, 12/13*p or 3/4p, 5/6p, 12/13*p	(3) 4/3
	*I. yaraligozi* Ünal, 2003	TR, Kastamonu prov., Yaraligoz Pass	sa	1*p, 3*p, 7*p, 11p	4
	*I. tosevski* Pavicevic, 1983	►MK, (1) Doiran lake near Nikolich vill.; (2) Mariovo range, Moklishte vill.	sa	3/4p, 5p, 6p, 7/8p, 12/13p	5
**4**	*I. taurica* Brunner von Wattenwyl, 1878	UA, S Crimea, Babugan Yayla plateau	sa	12/13p	1
	*I. gulae* Peshev, 1981►	BG, Yambol distr., Elhovo, 100 m	sm	2/3p, 12/13p	2
	*I. obtusa* Brunner von Wattenwyl, 1882►	BG, Lovech distr., C Stara Planina Mts, Pleven lodge	sa	2/3p, 12/13	2
	*I. camptoxypha* (Fieber, 1854) or *I. posthumoidalis* Bazyluk, 1971►	PL, Tatra Mts	sa	3/4p, 11/12p	2
	*I. altaica* Bey-Bienko, 1926►	RU, Altai Mts	sm	3p, 12/13	2
	*I. brunneri* (Fraivaldsky, 1867)	UA, Crimea, Chatyr Dag, 1250 m & Babugan Yayla	sm	7p, 9p	2
**5**	*I. modestior* Brunner von Wattenwyl, 1882	BG, (1) Vidin distr., Belogradchik; (2) Sofia distr., Vitosha Mt;	sa	3/4*p, 5/6p	2
		►SR, Novi Sad distr., near Kamenitsa vill		1/2p, 3/4*p	2
**6**	*I. kraussii* Brunner von Wattenwyl, 1878►	GR, Bavaria	sm	2p, 3/4p	2
	*I. pienensis* Maran, 1954►	PL, Bieszczady Mts	sm	1/2*p, 3/4p	2
**7**	*Isophya* sp.	TR, Erzurum prov., Ovit pass	sa	1/2*p, 10/12p	2
	*I. zernovi* Miram, 1938	TR, Artvin prov., near Kafkasor	sa	3*p, 11/12*p	2
	*I. autumnalis* Karabag, 1962	TR, Gümüşhane prov., Zigana pass	sa	2/4p, 10/12p	2
**8**	*I.* cf. *armena* Miram, 1938	TR, Sivas prov., Zara-Suşehri road	sa	3*p, 6p	2
	*I. schneideri* Brunner von Wattenwyl, 1878	TR, Ardahan prov., Ardahan-Çıldır	a	3p, 6p, 7p	3
**9**	*I. sureyai* Ramme, 1951	TR, Sivas prov., Zara-Suşehri road	sa	3p, 11/12p	2
	*I.* aff*. sureyai* Ramme, 1951	TR, Giresun prov., Tamdere	a	5/6p, 12/13p	2
	*I. speciosa* (Frivaldsky, 1867)	BG, ► (1) Rodope Mts & Stara Planina; (2) Byala	sa	1/2p, 3/4p	2
	*I. amplipennis* Brunner von Wattenwyl, 1878	TR, Bilecik prov., near Sögüt	sa	5/6p, 10p	2
	*I. rizeensis* Sevgili, 2003	TR, Rize prov., Ikizdere	a	3p, 5p, 11p	3
**10**	*I. major Brunner von Wattenwyl,* 1878	TR, Antalya prov., Kuruçay	sa	1/2p, 10/12p, Xp	3

BG = Bulgaria, GR = Germany, MK = Macedonia, PL = Poland, RU = Russia, SR = Serbia, TR = Turkey, UA = Ukraine; ►published by Grzywacz et al. 2011; X-type: a = acrocentric, sa = subacrocentric, sm = submetacentric; total = number of clusters in haploid genome; a slash between two numbers indicates imprecise identification of the chromosome pair (bivalent); p = paracentromeric; d = distal; * high or low activity between homologous chromosomes. The first column shows groups of species/phylogenetic lineages as suggested in published sources and/or inferred from unpublished own data: 1 – *I. straubei* group [5]; 2 – *I. rectipennis* group [5]; 3 – *I. modesta* group [5]; 4 – *I. pyrenaea* group [5]; 5 – *I. costata* group [5]; 6 – *I. kraussii* group [5]; 7 – *I. zernovi* group [46]; 8 – *I. schneideri* group [5,46]; 9 – *I. speciosa* group [5]; 10– *I. major* group [12].

### Cytogenetic mapping of ribosomal and telomeric DNA and NORs

**Figure 1:**
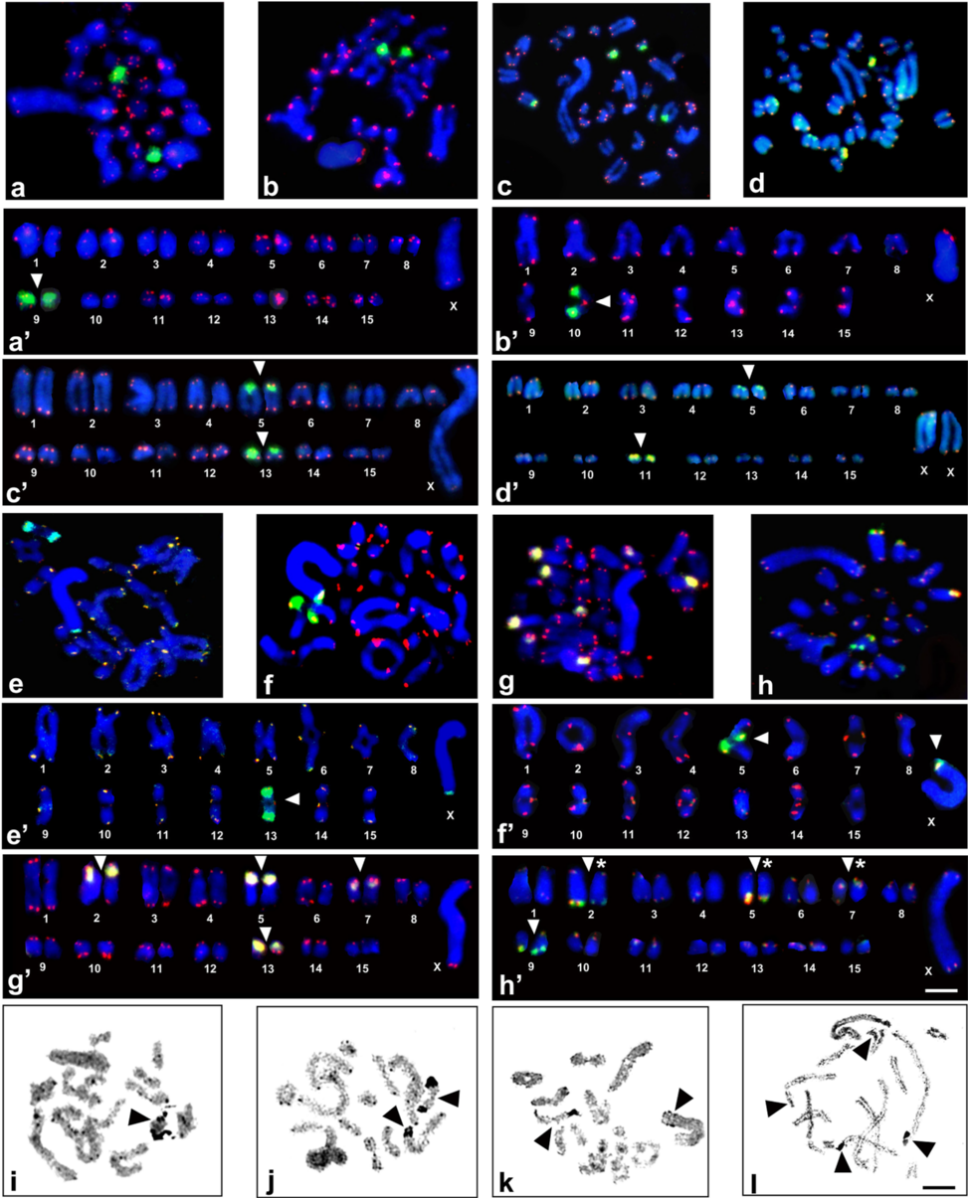
**Examples of FISH with both 18S rDNA (green) and telomeric DNA (red) probes in spermatogonial metaphase (a, c, d, g, h) or diakinesis (b, e, f), as well as karyotypes arranged from these divisions (a’–h’) and silver staining in diakinesis (i–k) and diplotene (l) of chromosomes for the following *Poecilimon *species: *P. cervus *(a, a’), *P. bosphoricus *(b, b’, i), *P. chopardi *(c, c’, j), *P. klisuriensis *(d, d’), *P. macedonicus *(e, e’, k), *P. ukrainicus/fussii *(f, f’), *P. affinis *(g, g’), and *P. zwicki *(h, h’, l).** White arrowheads point to rDNA clusters near the centromeric or distal regions of the chromosomes. Hybridization areas vary in size between some homologous chromosomes, which are marked with an asterisk (*). Black arrowheads indicate the presence of one **(i)**, two **(j, k)**, or four **(l)** active NORs. 18S rDNA signals in mitotic metaphase/diakinesis coincide with NORs. Bar = 10 μm.

**Figure 2:**
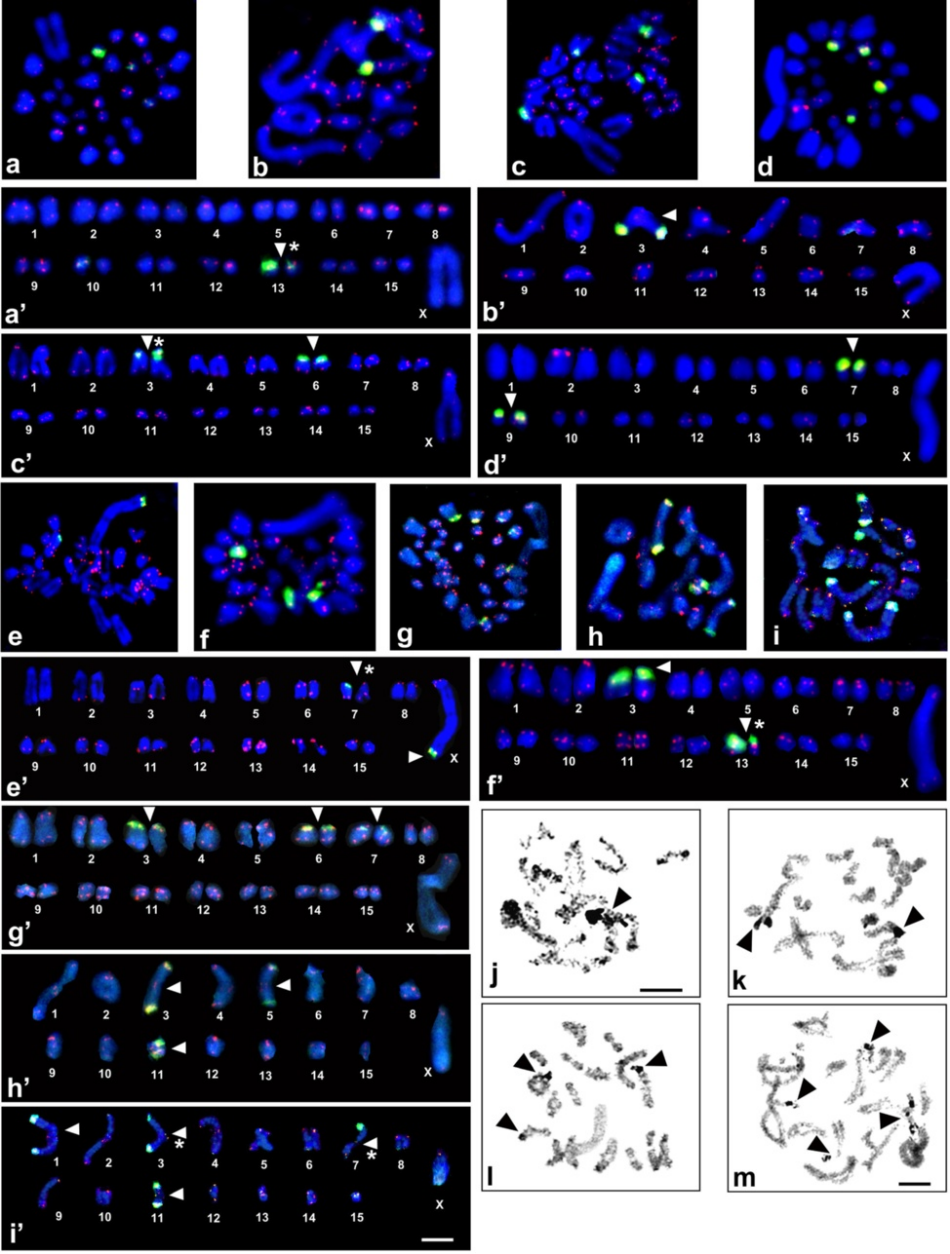
**Examples of FISH with both 18S rDNA (green) and telomeric DNA (red) probes in spermatogonial metaphases (a, c, d, e, f, g) or diakinesis (b, h, i) as well as karyotypes arranged from these divisions (a’–h’) and silver staining in diakinesis (l) and diplotene (j, k, m) of chromosomes for the following *Isophya *species: *I. thracica *(a, a’, j), *I. nervosa *(b, b’), *I. *cf. *armena *(c, c’, k), *I. brunneri *(d, d’), *I. rectipennis *– Bulgarian population (e, e’) and Turkish population (f, f’), *I. schneideri *(g, g’, l), *I. rizeensis *(h, h’), and *I. yaraligozi *(i, i’, m).** In the karyotypes, white arrowheads indicate the chromosomal location of rDNA clusters and an asterisk (*) marks differences in size between some homologous chromosomes **(a’, c’, e’, f’, i’)**. Black arrowheads indicate the presence of one **(j)**, two **(k)**, three **(l)**, or four **(l)** active NORs which coincide with 18S rDNA signals. Bar = 10 μm.

Cytogenetic maps of 18S rDNA were obtained for 39 *Poecilimon* taxa (Table 1) and 40 *Isophya* taxa (taken together with those previously published [20]) (Table 2). Representative hybridized metaphasic chromosomes or bivalents are shown in Figures 1a–h, 1a’–h’ and 2a–i, and 2a’–i’. In both genera, the number of rDNA sites per haploid genome ranged from one to five; they were located on the autosomes, and rarely on the sex chromosome – in *P. macedonicus* (Figure 1e), *P. marmaraensis* (not shown), *P. ukrainicus/fussii* (Figure 1f), and also *I. pavelii* ([20] – see Figure 2a), *I. major* (not shown), and one population of *I. rectipennis* (Figure 2e).

Most *Poecilimon* taxa carried one (50%) or two (45%) rDNA loci, and only two species exhibited four loci (5%). When a single rDNA cluster was detected, it was always located on a small pair near the paracentromeric region (one of the pairs 9–12; Figure 1a,b); only in *P. jonicus tessellatus* it appeared in the interstitial region (not shown). Two rDNA clusters were evident on long/medium and short chromosome pairs near the paracentromeric region (Figure 1c,d), except for *P. macedonicus* (distal location on the X chromosome; Figure 1e) and *P*. aff. *glandifer* (interstitially near its distal region, probably on the 3rd or 4th pair; not shown). FISH revealed rDNA on four bivalents only in *P. affinis* (in the paracentromeric region; Figure 1g) and *P. zwicki* (paracentromeric or distally located; Figure 1h). While the number of 18S rDNA loci varied from one to five in *Isophya*, most species showed two locations (62.7%); rarely one (11.8%), three (11.8%), four (7.8%), or five (5.9%) clusters (including polymorphism between populations in some species) (Figure 2a–i). Most 18S rDNA loci were situated in different-sized autosomes in the paracentromeric region, however distally to the centromere on a long pair (3) in *I. straubei* ssp. (not shown), as well as on the subacrocentric X chromosome and a medium-sized bivalent (pair 5) in *I. rectipennis* from both Bulgarian populations (Figure 2e and see Figure 2c,d in [20]).

Individuals from different populations demonstrated the same rDNA-FISH signal location in *Poecilimon brunneri*, *P. roseoviridis*, *P. macedonicus*, *P. schmidtii*, *Isophya miksici*, *I. longicaudata longicaudata*, *I. modestior* (from Bulgarian populations), *I. rhodopensis petkovi*, and *I. tosevski*. On the other hand, interpopulation variation in the rDNA signal pattern observed in *P. armeniacus* and *I. rectipennis* (Figure 2e,f), was also previously reported in *I. bureschi*, *I. rhodopensis rhodopensis*, and *I. rh. rhodopensis/I. rh. leonorae* intermediate forms [20] (Table 1 and 2). Sometimes the rDNA cluster varied in size between homologous chromosomes (Table 1 and 2, see the chromosome number marked with an asterisk; Figures 1’ and 2a’ ,c’ ,e’ ,f’ ,i’).

FISH with the (TTAGG)_*n*_ probe (tDNA-FISH) was used for spermatogonial or oogonial mitosis and spermatocyte nuclei at different stages of meiosis. In all species of both genera, tDNA-FISH signals were detected at the distal ends of most chromosomes, but showed variation in size and intensity on the autosomes and X chromosomes in some species (Figure 1a–h; 2a–i).

In all the species examined in the present study, rDNA-FISH signals were co-localized with the active NORs visualized by AgNO_3_ staining (Figures 1i–l, 2j–m). A lack of a full congruency between rDNA location and NOR activity has been previously reported for some autosomes and the X chromosome only in four *Isophya* species [20].

### Heterochromatin patterns revealed by banding techniques

**Figure 3:**
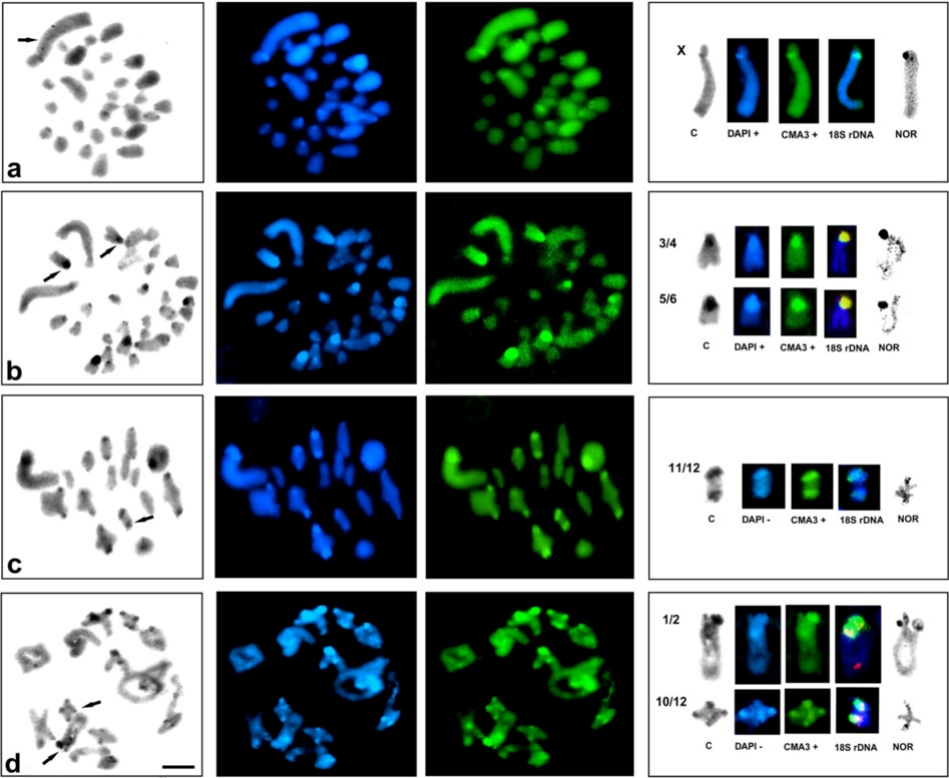
**C-, DAPI (blue), and CMA**_**3 **_**(green) stained heterochromatin and selected chromosomes (with C-bands, DAPI, CMA**_**3**_**, 18S rDNA, NOR) of spermatogonial metaphase in *I. major *(a) and *I. modestior *from the Bulgarian population (b) as well as of diplotene in *P. martinae *(c) and *Isophya *sp. (d).** In the selected chromosomes, C/DAPI/CMA_3_ blocks were located very close to each other, but bright CMA_3_ signals coincided with 18S rDNA and active NORs **(a,b)**; thick C-bands were DAPI-, whereas bright CMA_3_+ signals were co-localized with rDNA-FISH/NOR signals **(c,d)**; heteromorphism in the paracentromeric region of 1/2 in *Isophya* sp. **(d)** seems to be corroborated by all staining methods. The arrows (left panels) indicate selected chromosomes (right panels). Bar = 10 μm.

Table 3 shows variation among the analyzed species and genera using C–banding (only double/thick C–bands) and DAPI/CMA_3_ patterns. Heterochromatin blocks can be characterized as DAPI-/CMA_3_+ (GC-rich), DAPI+/ CMA_3_- (AT-rich) or DAPI+/CMA_3_+ (containing both AT- and GC-rich regions). Generally, thick C-bands on most chromosomes showed bright DAPI+ and CMA_3_+ signals in pericentromeric or, rarely, interstitial or distal regions. In such cases, DAPI and CMA_3_ blocks were located very close to each other, but only bright CMA_3_ signals coincided with 18S rDNA and active NORs (Figure 3a,b). In some species AT-bands were not detected (designated as DAPI- and marked as “0” in Table 2), while bright CMA_3_+ bands were co-localized with rDNA-FISH/NOR signals (Tables 1, 2, 3; Figure 3c–d). Unfortunately, it was not possible to separate DAPI/CMA_3_ regions to compare them with rDNA locations in *Poecilimon bischoffi*, *P. orbelicus*, *P.* aff. *ledereri*, and *P. marmaraensis*.

**Table 3:** **Heterochromatin patterns; localization of C-bands, DAPI and CMA**_**3 **_**signals in *Poecilimon *** **and *Isophya *** **species**

	**Species**	**Thick C-bands**	**Position of fluorochrome bands**	
			**DAPI+**	**CMA**_**3**_**+**
**1**	*P. ledereri*	most p	=C	most; **11/13**
	*P.* aff. *ledereri*	most p	=C	most;
	*P. orbelicus*	most	=C	most;
	*P. armeniacus* TR-Balik lake	all p	=C	all; **9/10**
	*P. armeniacus* TR-Camlibel pass	most p	=C	most; **11/12**
	*P. ampliatus*	2p + d, 3p, 4p, 9/10p	=C	**2, 9/10**
	*P. pechevi*	2/3d, 3/4p, 5p, 12/13p	=C	2/3, **3/4, 12/13**
	*P. ebneri*	X i, 2/3d, 3/4*p, 2/13p	most	X i, 2/3d, 3/4*, 12/13
	*P. klisuriensis*	most p	=C	**5, 11**
	*P. marmaraensis*	2/3d, most p	=C	most;
**2**	*P.* cf. *karakushi*	most p	=C	most; **11/13**
**3**	*P. cervus*	most p	=C	most; **3, 9**
	*P. bischoffi*	all p	=C	all
**4**	*P. chopardi*	most p	=C	most; **5, 13**
**5**	*P. brunneri*	most p	=C	most; **11/13**
	*P. macedonicus*	most p	=C	most; **13, X**
	*P. zwicki*	2/4d, 5d, 7p, 9/10 d	0	**2, 5, 7, 9**
**6**	*P. jonicus tessellatus*	1, 2, 3, 12/13 all i	=C	1, 2, 3; **12/13**
	*P. martinae*	most p	=C	most; **11/12**
**7**	*P. schmidtii*	3/4p, 6/7p	=C	**3/4, 6/7**
	*P. zonatus/varicornis*	0	0	**4/5, 10/13**
**8**	*P. ornatus*	most p	=C	most; **3/4, 12/13**
	*P. affinis*	all p	=C	all; **2, 5, 13**
**?**	*P. ataturki*	12/13p	0	**12/13**
	*P. celebi*	most p	=C	most; **2/3, 11/13**
	*P.* aff. *glandifer*	1-5p, 3/4d*	1-5	1-5, 3/4*, 10/12
**1**	*I. hospodar*	3, 10/12p	=C	**3, 10/12**
	*I. straubei* ssp.	2/3*p, 6p	0	3*, 6
**2**	*I. nervosa*	3/4p	3/4	**3**
	*I. thracica*	2, 12/13p	most	most; **13**
	*I. stenocauda obenbergeri*	3/4*p,10/11p,11/12*p	10/11,11/12	11/12*
**3**	*I. bureschi* BG-Plana	3, 4, 11/12, 13/14	=C	**11/12**
	*I. yaraligozi*	most p	=C	1*, 3*, 7*, 11
**4**	*I. taurica*	10/11, 12/13*	most	most; 12/13*
	*I. brunneri*	3i, 7p, 9p, 11/12p	0	**7, 9**, 11/12
**5**	*I. modestior* BG	3/4*p, 5/6p, 12/13p	4/5, 12/13	3/4*, 5/6
	*I. modestior* SR	1 i; 2/3p, 4p, 9p, 10/11p	0	**1/2, 3/4**
**7**	*Isophya* sp.	most p, 1/2*p	=C	1/2*, 10/12
	*I. zernovi*	3/4p, 10/12p	=C	**3, 11/12**
	*I. autumnalis*	0	0	**2/4, 10/12**
**8**	*I. schneideri*	3p, 5p, 11p	7/8	**3, 5, 11**
**9**	*I. sureyai*	3/4p, 11/12p	=C	**3/4, 11/12**
	*I. speciosa*	1/2p, 3/4p, 11/12p, 13/14p	0	**1/2, 3/4**
	*I. amplipennis*	2p, 5/6p, 10p	5/6, 10	2, **5/6, 10**
	*I. rizeensis*	2/3p, 6p, 7/8p	2/3	**3, 6, 7**
**10**	*I. major*	1/2p, 10/12p, X p	1/2, X	**1/2, 10/12, X**

A slash between two numbers indicates imprecise identification of the chromosome pair (bivalent); C = C-bands; 0 = DAPI+ signal not visualized; p = paracentromeric, i = interstitial, d = distal; X = sex chromosome; *presence of polymorphism in homologous chromosomes; pairs of chromosomes with CMA_3_+ marked by bold typeface coincide with 18S rDNA and NORs; TR = Turkey, BG = Bulgaria, SR = Serbia. The first column corresponds to that in Tables 1 and 2.

Some species in both studied genera exhibited heteromorphism in terms of rDNA-FISH signal size/strength and C/DAPI/CMA_3_ bands between homologue chromosomes or different-sized autosomes (as indicated with an asterisk in Tables 1, 2, 3); for example, in *P. zwicki* (Figure 1h’), *I. thracica* (Figure 2a’), *I.* cf. *armena* (Figure 2c’), *I. rectipennis* (Turkish and Bulgarian populations; Figure 2e’ ,f’), *I. yaraligozi* (Figure 2i’), and *Isophya* sp. (Figure 3d).

## Discussion

By mapping rDNA (and also potential NORs) and two heterochromatin classes we set out to determine whether these methods may be used to identify chromosome markers useful for studying the genomic organization and diversity of *Poecilimon* and *Isophya* and for distinguishing evolutionary lineages. The differences and similarities revealed between the two genera are listed below.

### Differences in genome organization revealed by cytogenetic markers

Given the stable karyotype in both genera (2n = 31), variation in the number of acrocentric chromosomes involved only the sex chromosomes. This could be due to the occurrence of a pericentric inversion that converted the original/ancestral acrocentric X chromosome to a subacro-/submetacentric one. These karyotype characteristics were found in 35 out of 42 *Isophya* taxa (present study; [19]), but in only three out of 39 *Poecilimon* species/subspecies (present study; [38]). Thus, changes in the morphology of the X chromosomes are common in *Isophya*, in contrast to *Poecilimon* and other Barbitistini [39].

Based on the number of rDNA clusters, the genus *Poecilimon* was divided into two main, almost equal, groups carrying the FISH signal on one or two chromosomes. In turn, the prevalent number of rDNA locations in *Isophya* was two, while either one or 3–5 locations occurred in some cases. Three rDNA clusters were usually connected with a large amount of heterochromatin in the chromosome set (also [19] for *Isophya*). A high number of NORs/rDNA loci with variable positions is characteristic of some other Barbitistini (*Barbitistes*, *Polysarcus*, *Phonochorion*; [37]), involving groups of species of recent origin. In both analyzed genera, most species had rDNA on one or two chromosomes, which makes it difficult, especially in *Poecilimon*, to assess their ancestral status. The presence of paracentromeric rDNA loci only on a single bivalent was previously observed in other Phaneropterinae species: the European *Odontura*[36] and *Phaneroptera falcata*[40], as well as in the African *Lunidia viridis*[34] and four species of the genus *Eurycorypha*[35]. In other Tettigoniidae, single rDNA-positive FISH signals have been observed in European representatives of Saginae and Bradyporinae [32,35]. In addition, one active NOR seems to be a typical feature of karyotypes with the ancestral chromosome number in Tettigoniinae [41].

Thick C/DAPI/CMA_3_ bands were observed on most chromosomes in *Poecilimon* suggesting the presence of more families of repetitive DNA sequences in this genus as compared to the heterochromatin patterns in *Isophya*. Thus, these bands appear to be a feature differentiating the karyotypes of the two genera (Table 3). A high amount of heterochromatin was earlier reported for some other genera of Barbitistini [37].

### Similarities in genome organization revealed by cytogenetic markers

All the studied species had an ancestral (for Tettigoniidae) diploid chromosome number, and thus the variability in the number and location of rDNA loci probably resulted from transposition involving mobile elements or ectopic recombination. Similar mechanisms have been suggested for Acrididae [25], Lepidoptera [24], and Scarabaeinae [23]. Generally, rDNA loci are coincident with active NORs and GC-rich heterochromatin, which indicates the presence of multiple repetitive DNA sequences. Some species in both genera showed different intensities of rDNA hybridization signals on homologous pairs of autosomes, and also heteromorphism in the pattern of heterochromatin distribution (indicated by an asterisk in Tables 1, 2, 3). These differences were detected consistently by all the banding techniques used and observed in those chromosomes in which heterochromatin occurred in large quantities (i.e. in the form of large paracentromeric heterochromatic blocks). Such intraspecific polymorphism may be the result of different mechanisms, i.e. tandem duplication of ribosomal genes, unequal meiotic crossing-over, translocation rearrangements, or homologous recombination [23,42,43].

Our results point to some general patterns in the structure of heterochromatin and NOR/18S rDNA locations: (1) DAPI and CMA_3_ staining cause bright fluorescence co-occurring with thick paracentromeric C-blocks, suggesting the presence of a high number of AT- and CG-base pairs; (2) sometimes CMA_3_ does not detect active NORs but a special type of GC-rich heterochromatin associated with this region [44]; (3) NOR/rDNA-FISH regions show a bright CMA_3_ signal; (4) the pattern of distribution of GC-rich blocks indicates some level of dynamism in the genome content of heterochromatin areas and may be related to specific changes characterizing groups of taxa. Thus, different heterochromatin types suggest the occurrence of specific rearrangements of repetitive DNA families that have evolved during the diversification of *Isophya* and *Poecilimon* and are characteristic of certain phylogroups.

### A comparison of cytogenetic and taxonomic traits

The evolution of repeated DNA families is dominated by genomic events such as duplication and spreading which may impede tracking the evolutionary history of the sequence, and thus prevent using these as genetic markers. However, in some cases we find concordance of cytogenetic data with phylogenetic traits such as morphology, bioacoustics, and molecular data. Previously published phylogenies [22] and speculations on species groupings using morphology and behavior are discussed below and summarized in Tables 1, 2, 3 (the first column). The European representatives of the *Poecilimon ampliatus* group (Table 1, group 1) *sensu stricto*[45] with monophyletic origin [22] show a more or less uniform location of two paracentromeric rDNA clusters on long and short autosome pairs, while the Anatolian species along with *P. orbelicus* (regarded as a monophyletic lineage except *P. armeniacus*[3]) exhibit only one FISH signal. This may provide additional data for refining species relationships by exploring correlations with morphological and behavioral traits [10,45]. Interesting examples deserving particular attention include the little known *P. glandifer* and *P. ataturki*, which were placed within the *P. ampliatus* group [46], possibly on account of having an abdominal tergal gland (a structure that is occasionally found within several groups of Barbitistini). And though their affinities have not been phylogenetically studied yet, there is some evidence (Chobanov et al., unpublished data) supporting their cytogenetic distinctness from group 1. An example of concordance of the present data with morphological, behavioral, and molecular traits is group 2, including taxa of the *P. syriacus* group [14]. Two groups of sibling species are divided between one and two rDNA-FISH signal positions – the *P. bosphoricus* group (group 3) [17] and the *P. brunneri* group (group 5) [10,22]. No proposed relationships between these were reflected in the chromosome markers, except for the phylogenetic affinities of representatives of the *P. bosphoricus* group with one chromosome rDNA location (*P. miramae* + *P. cervus* + *P. heinrichi* + *P. bosphoricus*), which are polyphyletic with respect to those with two locations (*P. anatolicus* and *P. turcicus*), according to mtDNA data [22]. The four rDNA clusters in *P. zwicki* (the last being a basal member of the *P. brunneri* group according to molecular data [22]) and *P. affinis* probably represent autapomorphies.

The genus *Isophya* shows a more complicated pattern of rDNA/heterochromatin organization than *Poecilimon*. In most taxa, two 18S rDNA sequences were located on two chromosome pairs. This seems to be a plesiomorphic state for the group, characteristic of the most primitive lineages [5], for example, *I. hospodar, I. straubei* (Table 2, group 1), and *I. rectipennis* (group 2). Yet, all of these exhibit quite a peculiar location of the studied markers. In some taxa, a significant variation of chromosome markers (Tables 2, 3) corresponds to distinct intraspecific and intrapopulation genetic and/or morphoacoustic variation [5,21,47]. Three to five FISH signal-positive chromosomes have been found in taxa of recent origin and/or populations where hybridization by secondary contact of haplotypes is suspected. The latter case concerns the *I. modesta* group of species [5,19], and especially the *I. rhodopensis* complex, representing a few subspecies inhabiting a large area of secondary contact between possibly formerly isolated populations, where intrapopulation variation in the number of rDNA clusters is observed. This is usually connected with ongoing speciation and hybridization in zones of secondary contact [5,21,47].

According to our results analyzed in conjunction with known systematic and phylogenetic data transposition and recombination, or, alternatively, occasional loss of rDNA fragments clusters, may have often occurred multiple times in different lineages of *Isophya* and, rarely, in *Poecilimon*. Similar events resulting in a variable heterochromatin structure have been observed in other groups of Barbitistini, mostly taking place within or between taxa of recent origin [37].

## Conclusions

The cytogenetic study presented herein constitutes the next step towards a better understanding of chromosomal organization and evolution within Phaneropterinae. We have outlined some general tendencies of chromosomal organization within *Isophya* and *Poecilimon*. These may result in unique species-specific characters or involve homoplastic changes in distinct lineages. And although chromosomal distribution of repeated DNA sequences could represent intrinsic aspects of the evolutionary dynamics of the repeated DNA families, our results have sometimes reflected inferences based on morphological, behavioral, and/or gene-sequence data. Thus, mapping of rDNA sequences and heterochromatin may in some cases be used as an additional marker for understanding relationships and routes of speciation within Barbitistini.

## Material and methods

A total of 95 specimens of 39 *Poecilimon* species/subspecies and 112 specimens of 40 *Isophya* taxa (including 21 previously described species/subspecies [20]) were studied. Male adults and nymphs and female nymphs were collected from 2006 to 2012 in Eastern Europe and Turkey. Details of taxon names and their possible grouping and collection sites are given in Tables 1 and 2. Chromosome preparations were obtained from the gonads of last instar nymphs or adults. Testes and ovarioles were incubated in hypotonic solution (0.9% sodium citrate), fixed in modified Carnoy’s solution – ethanol: acetic acid (3: 1), and stored at 2°C until use. Chromosome preparations for the examination of nucleolus organizer regions (NORs) and fluorescence *in situ* hybridization (FISH) experiments were made by tissue squashing using 45% acetic acid, subsequent removal of cover slips by the dry ice technique, and air-drying. The silver staining method (AgNO_3_) for NOR location was performed according to the protocol by Warchałowska-Śliwa and Maryańska-Nadachowska [48]. Constitutive heterochromatin was revealed by the C-banding technique as described by Sumner [49]. In order to identify GC- and AT-rich regions, the preparations were stained with CMA_3_ and DAPI, respectively [50].

FISH with ribosomal 18S DNA (rDNA) genes and the telomeric sequence (TTAGG)_*n*_ was performed exactly as described in Warchałowska-Śliwa *et al.*[32]. Preparations were counterstained with DAPI (4,6-diamidino-2-phenylindole) and mounted in an anti-fade medium with DABCO. Images for FISH were captured using an AXIOSCOP 2 (Zeiss) microscope equipped with a CCD camera, filter set, and an ISIS5 image processing package (Metasystems GmbH) at the Microscopic Centre of the Institute of Cytology and Genetics, SB RAS, Novosibirsk, Russia. Slides with silver staining impregnation were examined under a Nikon Eclipse 400 light microscope fitted with a CCD DS-U1 camera and an NIS-Elements BR 3.0 image analyzing system (Nikon). At least 10 meiotic divisions (from diplotene to metaphase I) per male and at least three spermatogonial and/or oogonial metaphases (for some specimens) were analyzed using three techniques: FISH, AgNO_3_ staining, and classical cytogenetic methods (C-banding and DAPI/CMA_3_ staining). In each species, both the rDNA-FISH pattern and the location of active NORs were always recorded on meiotic bivalents in prophase I in the same individuals.

## Abbreviations

FISH: Fluorescence *in situ* hybridization; CMA3: Chromomycin A_3_; DAPI: 4’6 diamidino-2- phenylindole; NOR: Nucleolar organizer region; tDNA-FISH: Telomeric probe; rDNA: Ribosomal DNA; BG: Bulgaria; GR: Germany; MK: Macedonia; PL: Poland; RU: Russia; SR: Serbia; TR: Turkey; UA: Ukraine; SL: Slovenia; a: acrocentric; sa: subacrocentric; sm: submetacentric; p: paracentromeric; d: distal; i: interstitial; i–d: interstitial near the distal end; C: C–bands; 0: DAPI + signal not visualized.

## Competing interests

The authors declare that they have no competing interests.

## Authors’ contributions

BG, DPCH, EW-Ś made major contributions to conception and design of analyses, interpretation of data and writing the manuscript. AM-N, TVK, K-GH took part in data analysis. All authors interpreted the results, have read and approved the final manuscript.
